# Exploring the role of ascending aortic length to prevent type A acute aortic dissection

**DOI:** 10.3389/fcvm.2026.1869551

**Published:** 2026-09-04

**Authors:** Alberto Pozzoli, Livia Waltert, Mirko Muretti, Laura Anna Leo, Marta Sannito, N'Guessan Kra, Tiziano Torre, Enrico Ferrari, Susanna Grego, Stefanos Demertzis

**Affiliations:** 1 Heart Surgery Unit, Cardiocentro Ticino Institute, EOC, Lugano, Switzerland; 2 Faculty of Biomedical Sciences, Università Della Svizzera Italiana (USI), Lugano, Switzerland; 3 Cardiovascular Imaging & Cardiology Unit, Cardiocentro Ticino Institute, EOC, Lugano, Switzerland; 4 Faculty of Medicine, University of Zurich (UZH), Zurich, Switzerland; 5 Rare Cardiovascular Disease Unit, Cardiocentro Ticino Institute, EOC, Lugano, Switzerland; 6 Faculty of Medicine, University of Bern, Bern, Switzerland

**Keywords:** acute aortic dissection risk, aortic diameter/height index, ascending aorta aneurysm, ascending aortic length, prevention, risk-reduction

## Abstract

**Background:**

Stanford type A acute aortic dissection (AAD) often occurs before the given threshold for prophylactic surgery. This study investigates the role of the ascending aortic length (AAL) integrated to aortic height index (AHI) as an additional factor in the risk stratification of AAD. Thereby, the relationship between AAL and the aortic diameter, as well as the patient's height, will be evaluated.

**Methods:**

Patients undergoing surgery for AAD at our Institution between January 2013 and February 2024 were prospectively collected and retrospectively analysed. The size of the aortic organ was retrospectively measured using computed tomography to define the maximum diameter and the length of the ascending segment by means of syngo.via. The AHI and AHI + length (AHIL) were calculated and compared in all patients. A control group without known aortic pathology had their ascending aorta studied using magnetic resonance imaging, including those with a moderate risk of dissection (AHI >= 2.9 cm/m).

**Results:**

During the study period, 119 patients were included. The mean aortic diameter at the time of diagnosis was 5.07 ± 0.6 cm, the mean AAL was 11.94 ± 2.23 cm. In the AHI probability density function, the peak for dissection was 2.94 ± 0.36 cm/m, while for AHIL was 9.8 ± 1.15 cm/m. The difference between the AHI and the AHIL in the risk categorization was highly significant (*p* < 0.0001). In 12 high-risk patients with aortic enlargement, from a cohort of 6,990 individuals with non-aortic MRI indications, the inclusion of AAL to AHI also changed the categorization risk (*p* < 0.002).

**Conclusion:**

The addition of ascending aortic length to the aortic height index provides a potentially useful exploratory morphologic index available to the cardiologic community, helping to discriminate individuals within the moderate risk for acute aortic events. Patients with an AHIL exceeding 9.7 cm/m should be referred to an Aortic Center for a proper multidisciplinary risk evaluation.

## Introduction

Acute ascending aortic dissection (AAAD) – namely Stanford type A aortic dissection - is a fatal disorder with an incidence of around 6 out of 100,000 people per year (1). The mortality rate for AAAD is around 1%–2% per hour in the first 24 h and over 75% within the first 2 weeks without treatment (1). Therefore, early detection and rapid treatment are crucial to increase survival chances (2). The AAAD often arises from a dilated ascending aorta (AAo), which is very often asymptomatic and its diagnosis frequently happens by chance. Thus, it is difficult to know its real prevalence in the general population and identify individuals at risk. In routine clinical practice, once an aortic aneurysm has been diagnosed, it should be monitored by computed tomography (CT) imaging at regular intervals until surgery is indicated (3, 4). This notwithstanding, type A aortic dissection often occurs before reaching the diameter threshold of 5.5 cm, at which surgery is indicated prophylactically (3, 4). During the last years, many size and nonsize criteria have been proposed to predict the evolution of moderately dilated aortas (5). Remarkably, Zafar et al. presented in 2018 the Aortic Height Index (AHI), which simply indexed the aortic diameter to patient's height (6). Our institutional data confirmed that regardless of absolute aortic diameters, patients with an AHI exceeding 2.9 cm/m should ideally be referred to an Aortic Center for multidisciplinary risk assessment (7). The AHI is indeed a simple and effective tool to orient decision-making, thus helping to prevent aortic complications (Table 1). The former Aortic Size Index (ASI) was an aortic size/body surface area including patient's weight, which has been judged less accurate (5, 8–10). Further analysis recently revealed ascending aortic length (AAL) as predictor of aortic adverse events (AAEs), presenting a 5-fold increased risk/year if the length exceeds 13 cm, recommending a multidisciplinary evaluation already at an AAL of 11 cm (11). An even more powerful approach has been proposed by Wu et al., adding the AAL to the AHI: the new parameter is then AHI plus length divided by the patient's height (AHIL) (12). The aim of this study is to evaluate the impact of AHIL in *a posteriori* risk stratification of our AAAD patients and to assess its relevance to prevent adverse events in patients with asymptomatic aortic dilatation.

**Table 1:** Evolution of the most adopted indices and parameters to assess aortic risk.

Parameter	Calculation	Recommended threshold for surgical intervention	Image
Diameter	Diameter	5.5 cm	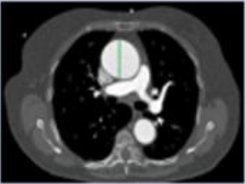
Aortic size index (ASI)	Diameter/BSA	>2.75 cm/m²	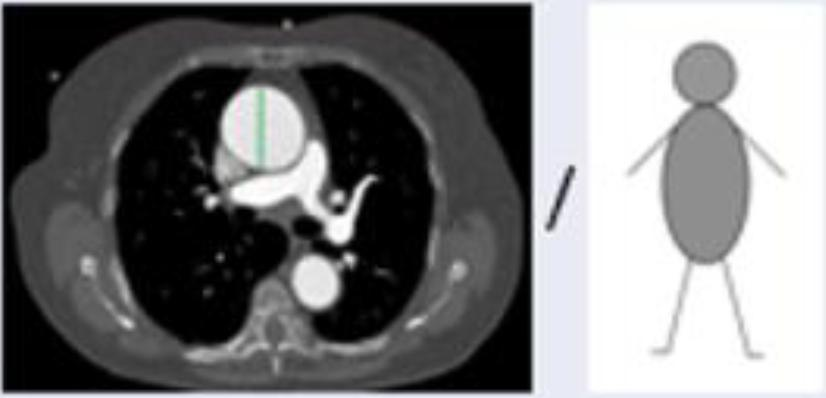
Cross-sectional area index	Cross-sectional area/height	>10 cm²/m	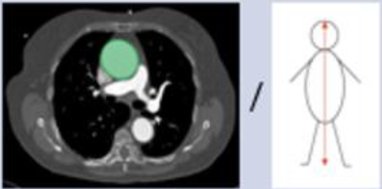
Aortic height index (AHI)	Diameter/height	Severe risk: > 4.1 cm/m High risk: 3.21–4.06 cm/m Moderate risk: 2.44–3.17 cm/m Low risk: <2.43 cm/m	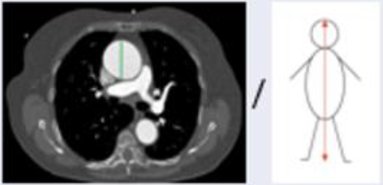
Ascending aortic length (AAL)	Length	>11 cm	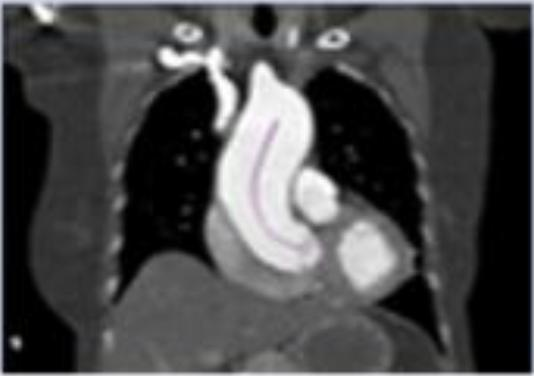
Aortic height index + length (AHIL)	(Diameter + length)/height	Severe risk: ≥12.57 cm/m High risk: 10.86–12.5 cm/m Moderate risk: 9.38–10.81 cm/m Low risk: <9.33 cm/m	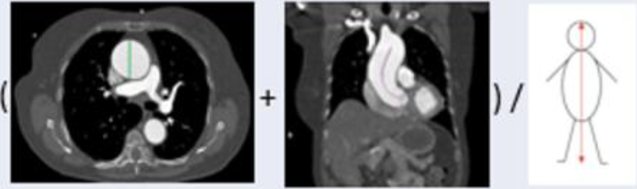

## Methods

### Hypothesis and study design

The objective of this single-center study is to determine whether AAL can serve as an additional predictive parameter for aortic disease progression and decision-making, by studying its relationship with AHI and patient's height.

In order to perform the analyses, the following two cohorts were selected:

1. “Study Group”: it has been prospectively collected and retrospectively analyzed. Consecutive patients with type A AAD who underwent surgical correction at our Institute between January 2013 and February 2024 were enrolled. Patients with known connective tissue disorders or significant aortic root dilation (more malignant patients with hypothetical unknown connective tissue disorders) have been excluded from the analyses.
2. “High-Risk Control Group”: it derives from a general population living in the Canton of Ticino undergoing magnetic resonance imaging (MRI) at our Institute for non-aortic indications, without any known aortic pathology. From this data collection, only patients considered at high-risk (AHI ≥ 29 mm/m) were selected and defined as “High-Risk Control Group”. This control group was selected with the purpose to allow a closer aortic comparison in terms of aortic diameter and aortic length in respect to the dissected “Study Group”.
3. This general population MRI database was prospectively collected from February 2013 to November 2023, with the aortic measurements obtained in each patient by a dedicated Cardiologist within the institutional MRI Unit (A.L.L.).

### Radiologic measurement of aortic dimensions

**Figure 1:**
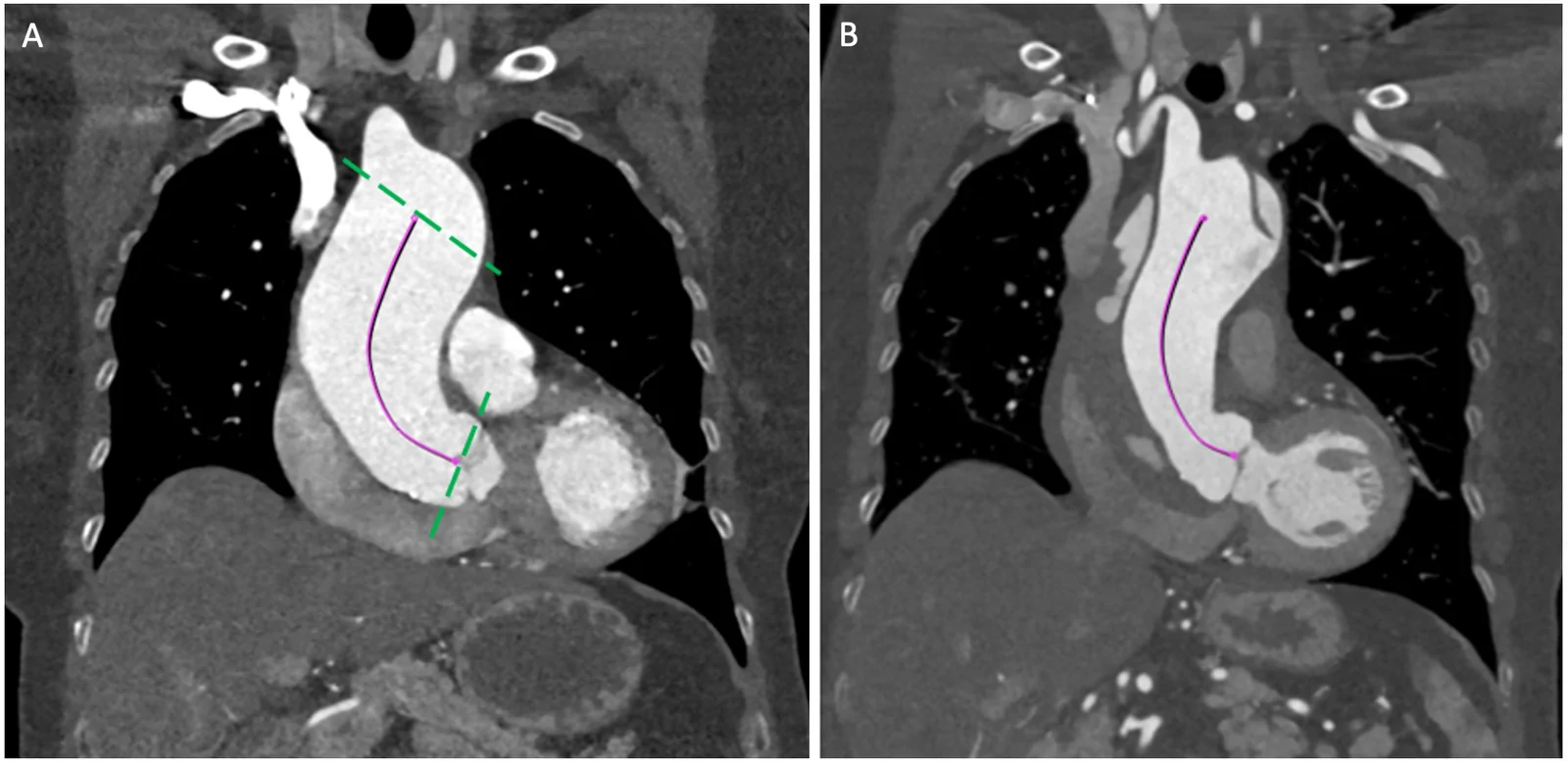
The AAL automatic measurements in multislice computed tomography angiography (CTA) were performed along the software-generated central line (purple) with syngo.via (siemens AG, Germany). Panel A. The AAL is measured with syngo.via from the aortic annular plane (proximal dotted green line) to the origin of the brachiocephalic artery (distal dotted green line). Prior to acute aortic dissection AAL was equal to 97.8 mm. Panel B. The AAL after acute aortic dissection incremented to 98.4 mm. AAL: ascending aortic length.

**Figure 2:**
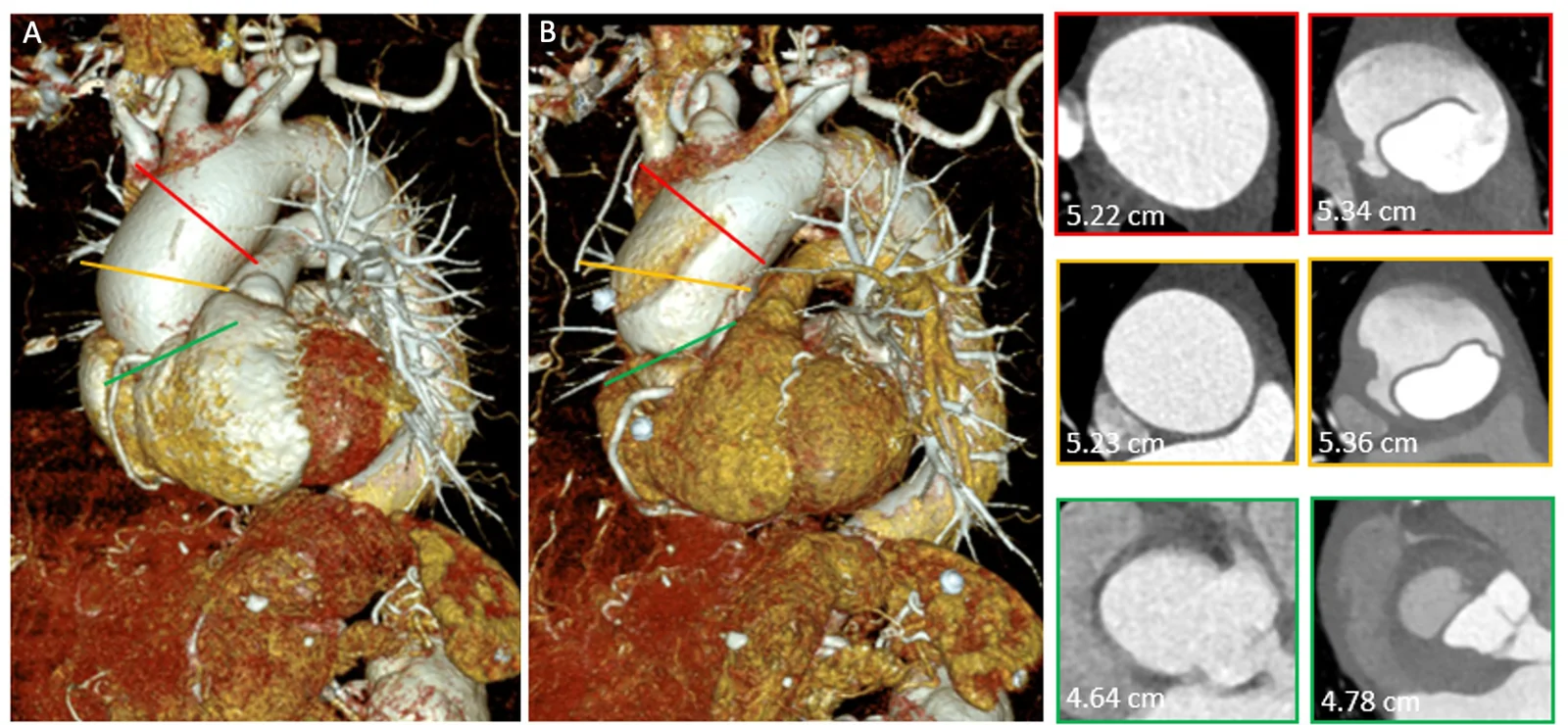
Panel A and B: Pre- and post-acute aortic dissection computed tomography angiography (CTA) imaging, revealing the morphological and geometrical changes at different segmental aortic planes during the dissecting process (aortic root, green line; mid-ascending aorta, yellow line; transition from the distal ascending aorta to the proximal aortic arch, red line).

1. In the “Study Group”, the maximum external diameter of the aortic organ, involving true and false lumen, and the AAL were recalculated for each patient using the preoperative CT scan (Figure 1). This was accomplished using maximum intensity projections (MIPs), which are two-dimensional reconstructions in CT imaging. Automatic measurements in CT vascular imaging were performed along the software-generated central line with syngo.via (Siemens AG, Germany). In particular, ascending aortic length is measured with syngo.via from the aortic annulus to the origin of the brachiocephalic artery (Figures 1, 2). All diameter measurements were doubly confirmed by the author (L.W.) and 3 senior team members (A.P., A.L.L., M.S.). The CT reports by the radiologists from the department of radiology were reviewed as a reference to ascertain that there was no obvious disagreement.
2. In the “High-Risk Control Group”, one single operator (A.L.L) performed all MRI examinations within the same imaging team, and each patient was evaluated separately. The aortic dimensions were measured equal to the CT scans, using the MRI sequences “T1 Vibe Caipirinha fs Cor” and “T1 Caipirinha fs Sag”. Ten measurements were taken by hand, and the average was determined, as no automatic central line was available for MRI imaging with syngo.via. The height of the Control Group was also determined as part of this examination.

### Assessment of aortic risk using AHI, AAL and AHIL

a) The assessment of aortic risk (major complications, aortic dissection, rupture, and death) was performed according to AHI's four categories (6): low risk ≤ 2.43 cm/m; moderate risk from 2.44 to 3.17 cm/m; high-risk from 3.21 to 4.06 cm/m; and finally, very high-risk ≥ 4.1 cm/m. The AAL parameter was used as an additional predictive aortic parameter, added to the AHI score to calculate the new AHIL score (Table 1).
b) The assessment of aortic risk was then recalculated according to AHIL's four categories (12): low risk ≤ 9.33 cm/m; moderate risk from 9.38 to 10.81 cm/m; high risk from 10.86 to 12.5 cm/m; and finally, severe risk ≥ 12.57 cm/m.

### Clinical data collection

Data collection of patients in the Study Group was derived from the institutional database, including pathological and family history, previous imaging assessment, and hypertension history. Height has been obtained from patients' charts, from the same examination. All data from operated patients were retrieved from institutional databases, and biometric parameters and the prevalence of cardiovascular risk factors in the general population of the Canton Ticino were extrapolated from reports of the Swiss Federal Statistical Office.

### Ethical committee

The **Project ID** was approved by the local ethical committee of Ticino under protocol **CE 3121, BASEC 2016-01812**. The written patient consent was waived due to the emergency setting inherent to this research study. In accordance to the World Medical Association (WMA) Declaration of Taipei, the local ethical committee of Ticino approved the collection, storage, and monitoring of the use of health data related to this Project, thus protecting individuals' rights, dignity, autonomy, privacy, and confidentiality in research involving their data.

### Statistical analysis

Statistical analysis was carried out using the Python scripting language, with some small R grafts for greater statistical accuracy. Python is supported by some libraries developed for data analysis and data representation such as Seaborn (13). To reduce outliers the Flooring and Capping technique was applied. First, using the interquartile deviation, the data were analyzed in general terms to show their distribution, and outliers that altered the normal distribution were highlighted using boxplots. Subsequently, the Flooring and Capping strategy was applied, i.e., setting minimum and maximum limits to the considerable values. The exploratory survey adopted simple statistical techniques and graphical representation of the data. For each representation, in addition to the control group data, the graphs and tables were divided by gender and risk level according to the AHI. Two-dimensional scatter plots were used, adding a third dimension, according to a third variable (categorical, in most cases the level of risk), generated by different colored points. A correlation analysis was also carried out, integrating three different approaches (Pearson, Spearman, and Kendal). Subsequent statistical analyses were performed using STATA version 13.8/Mac. The sign test, a non-parametric method, was used to assess whether the risk classification based on AHI was significantly different from the classification of AHIL of the aorta. This test was selected due to its robustness and suitability for smaller sample sizes as well as for data that may not follow a normal distribution, while also accepting the original scale of the risk category.

## Results

### Study group clinical characteristics

From January 2013 to February 2024, 119 patients with Stanford type-A AAD underwent surgery at our Institution, with the demographic characteristics shown in Table 2. The mean age at the time of surgery was 67 ± 12 years, and the mean height was 1.72 ± 0.1 m. Male sex was predominant (81 males, 68.07%). These patients represent the known living population with AAAD among 350.000 inhabitants in the Canton Ticino. The incidence of AAAD has been 2.72/100.000/per year. A dilation of the ascending aorta was known at previous imaging in 6/119 (5%). A history of hypertension was present in 85/119 (71.4%). Bicuspid morphology was represented in 7/119 (5.8%).

**Table 2:** Demographics of the study group and the control group. Demographics of the “Study Group” (119 patients diagnosed with AAAD) and of the “Control Group” (12 patients) without any known aortic disease who had their ascending aorta measured at MRI scans. AAAD: acute ascending aortic dissection; n/a: not applicable.

Parameters	Study Group (*n* = 119)	High-Risk Control Group (*n* = 12)
Age, years	66.6 ± 12.1	70.7 ± 10.9
Male, *n* (%)	81 (68%)	10 (83%)
Height (m)	1.72 ± 0.1	1.69 ± 0.09
Weight (Kg)	79.6 ± 17.6	78 ± 13.9
Body Mass Index	26.6 ± 4.79	27.9 ± 4
Body Surface Area	1.92 ± 0.24	1.9 ± 0.2
Hypertension, *n* (%)	85 (71.4%)	9 (75%)
Overweight, *n* (%)	74 (62.2%)	9 (75%)
Bicuspid aortic valve, *n* (%)	7 (5.9%)	n/a
Ascending Aortic Diameter (cm)	5.07 ± 0.6	5.03 ± 0.65
Ascending Aortic Length (cm)	11.94 ± 2.23	11.35 ± 1.57
AHI cm/m	2.94 ± 0.36	2.97 ± 0.51
AHIL cm/m	9.8 ± 1.15	9.69 ± 1.05
Stanford type A aortic dissection, *n* (%)	94 (79%)	n/a
Emergent Surgery, *n* (%)	111 (93.3%)	n/a
Post junctional ascending aorta replacement, *n* (%)	74 (62.2%)	n/a
Aortic valve replacement (including Bentall), *n* (%)	71 (59.7%)	n/a
Aortic hemiarch replacement, *n* (%)	81 (68%)	n/a
Hospital duration, days	16 ± 9.21	n/a
Intra-Hospital Mortality, *n* (%)	16 (13.45%)	n/a
Late death: more than 30 days out of hospital, *n* (%)	12 (10.1%)	n/a

### AHI and AHIL distribution compared in the study group

**Figure 3:**
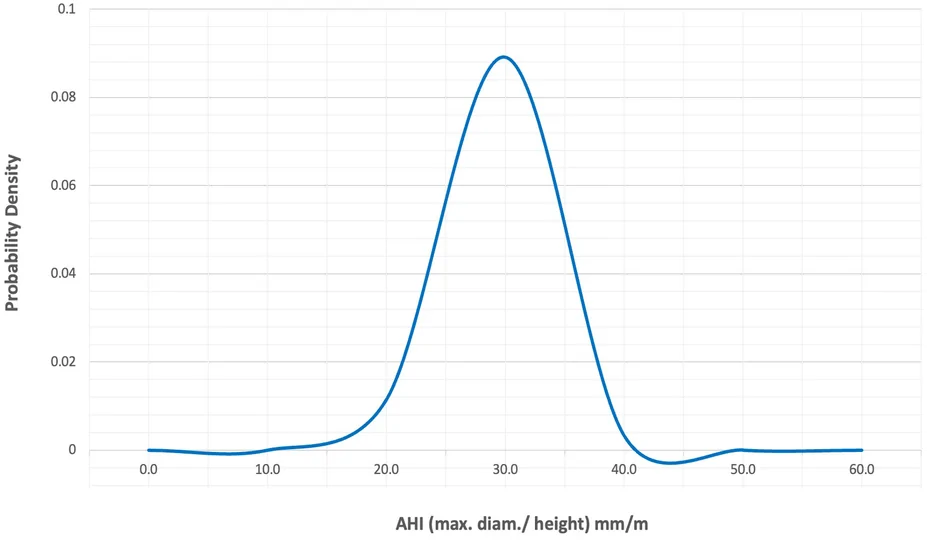
Aortic diameter/height index (AHI) of the “Study Group” patients, according to probability density (%).

**Figure 4:**
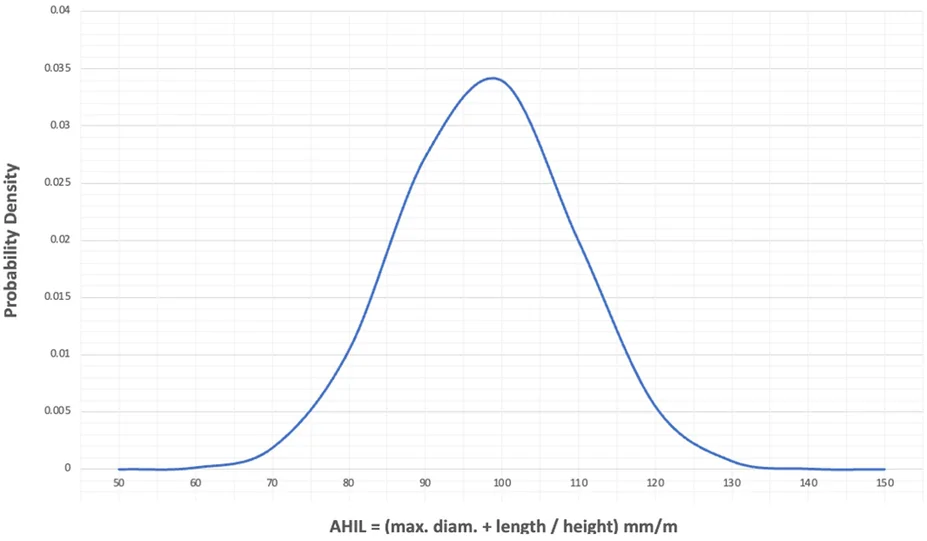
Maximal aortic diameter + length/height index (AHIL) of the “Study Group” patients, according to probability density (%).

**Figure 5:**
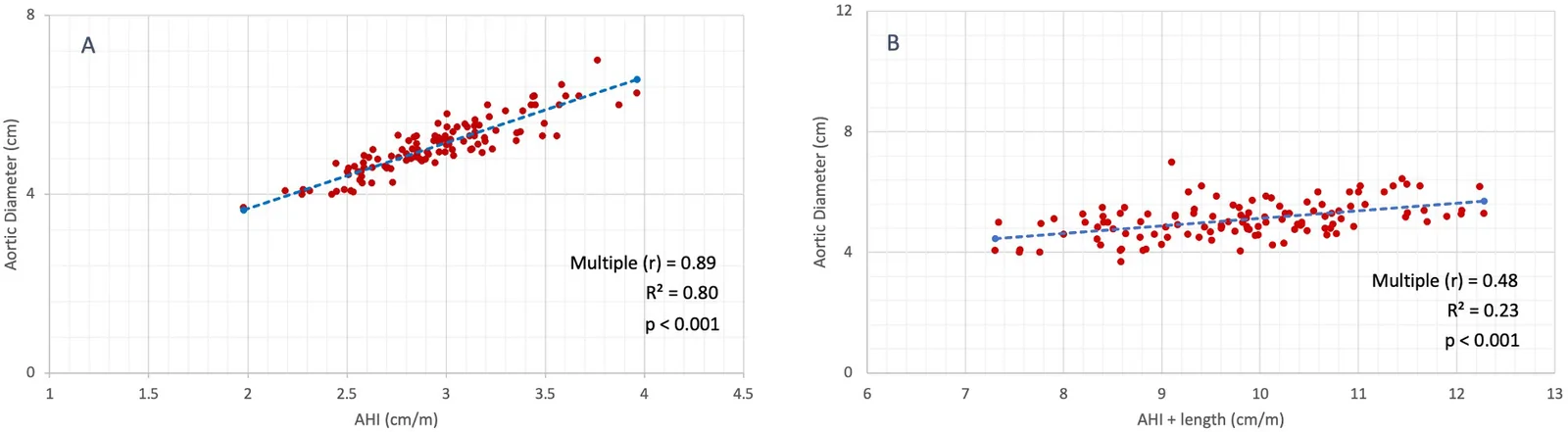
Panel A: linear correlation between aortic diameter/height index (AHI) and diameter of the ascending aorta in the “Study Group” patients. Panel B: linear correlation between AHI + length/height (AHIL) and maximal diameter of the ascending aorta in the “Study Group” patients. Correlation parameters are embedded in the two panels.

The retrospective calculation of the AHI showed the following distribution: 6/119 (5%) were considered low-risk, 83/119 (69.7%) moderate-risk, 26/119 (21.9%) high-risk, and 4/119 (3.4%) severe-risk for acute aortic events (Figure 3). At the time of the preoperative radiological diagnosis, the mean value of the maximum diameter of the ascending aorta was 5.07 ± 0.6 cm, and the mean value of the maximum length of the aorta was 11.94 ± 2.23 cm (Table 2). Instead, the calculation of AHIL revealed that 53/119 (44.5%) were at moderate-risk, 22/119 (18.5%) at high-risk, and 1/119 (0.9%) at severe-risk, categorized according to the AHI nomogram from Wu et al. (12). At the probability density function, the study group showed an AHI peak for dissection of 2.94 ± 0.36 cm/m, while for AHIL was 9.8 ± 1.15 cm/m (Figures 3, 4). When the AHI was compared to the AHIL for the capability of risk stratification for acute aortic events, the difference between the AHI and AHIL was highly significant (*p*-value < 0.0001). A linear correlation between AHI and the diameter of the ascending aorta was documented in the “Study Group” patients (Figure 5, Panel A). Specifically, the Pearson coefficient (multiple) r was equal to 0.891, R² = 0.8, 95% confidence intervals (CI) 0.849–0.920, with a *p*-value < 0.001. The regression slope was equal to 0.540, 95% CI (0.489–0.591), *p* < 0.001. The intercept of the regression was equal to 0.207, 95% CI (−0.054; 0.469), *p*-value = 0.119. Instead, the linear correlation between AHIL and the diameter of the ascending aorta was explored in the same patients' population (Figure 5, Panel B), showing a a correlation coefficients Pearson (multiple) r = 0.48 and a R² = 0.23 with 95% CI (0.326–0.608), with a *p*-value < 0.001. The regression slope was equal to 0.916, 95% CI (0.605–1.226), *p* < 0,001. The intercept of the regression was equal to 5.143, 95% CI (3.556–6.729), *p*-value < 0.001. The overall regression was statistically significant (*p* < 0.001).

**Figure 6:**
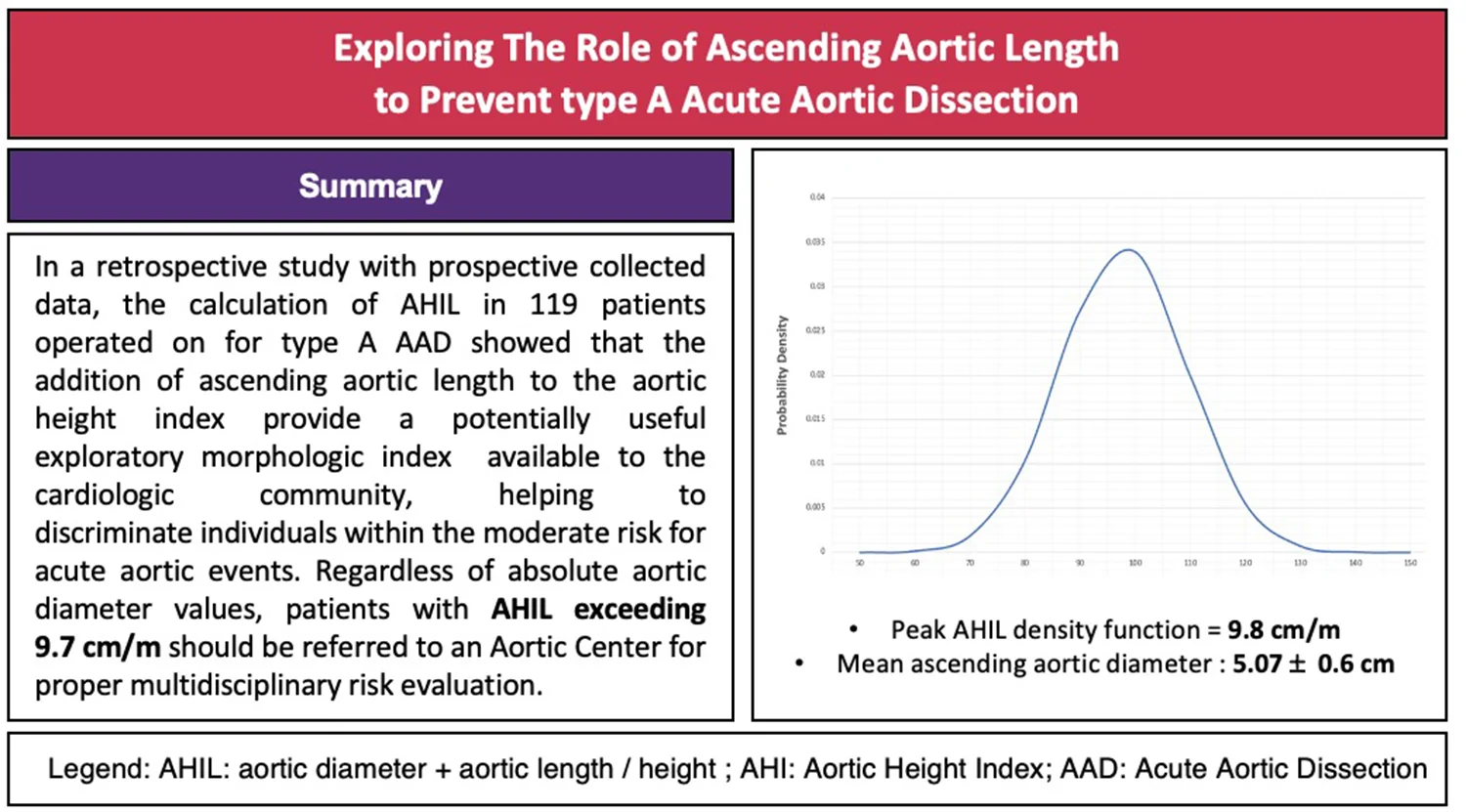
Central image: summary of the study and outcomes of the “study group”.

### AHI and AHIL distribution analysis in the high-risk control group

The Control group served to calibrate the two indexes in pure aneurysmatic aortas, in which the results of AHI calculation on the 6,990 subjects receiving cardiac MRI for non-aortic indications were as follows: a total of 377 (5.39%) persons presented at least a moderate risk for an acute aortic event (AHI range > 2.4 cm/m). Demographic data are detailed below in Table 2. Of these 377 patients, 12 patients (3.2%) were found to be at increased risk for acute aortic events (AHI > 2.9 cm/m). In this high-risk control group, the AHI was 2.97 ± 0.51 cm/m, the mean aortic length was 11.35 ± 1.57 cm/m, while the mean AHIL was 9.69 ± 1.05 cm/m (Table 2).

## Discussion

The integration of the aortic length into the aortic height index (AHIL) proved to be a refined prevention tool to detect aortic risk, when compared to AHI alone. The AHIL threshold values in a selected cohort of patients with aortic dissection are basically unreported in the Swiss and European population. The current study, performed on a selected number of dissected patients, should be interpreted in light of a stable cohort of patients who have had acute aortic event within a clearly defined region (Canton of Ticino), within a steady population of 350.000 people whose biometric parameters are available thanks to the Cantonal Statistics Office (USTAT). Importantly, at a federal level, these demographic statistics reflect the Swiss population. Therefore, the evidence of our contribution on the impact of aortic length and AHIL to improve prevention of acute aortic events, conveys three relevant key messages:

1. The largest group of patients identified by AHIL is the one at moderate risk, in which it becomes essential to have a threshold value for dissection. In our cohort, the “rupture” value occurs with an AHIL of 9.8 cm/m (Figure 4), which superimposes the one reported in the American cohort of New Heaven (12). This value is slightly overestimated due to the dissective process but is of great significance because it helps to discriminate the aortic risk within the moderate-risk group when AHIL is adopted.
2. The addition of ascending aortic length to the aortic height index significantly changed the categorization risk for acute aortic events, in respect to aortic height index alone (*p* < 0.0001). The AHIL is an index based on two parameters (aortic diameter and aortic length) normalize for the height, whereas the AHI is a single parameter (aortic diameter) normalized for the patient's height. Theoretically, two parameters should provide an assessment approximating a volumetric one, itself closer to the optimal morphological analysis of the aorta. However, when the correlation coefficients of the linear regression between AHI and aortic diameter were evaluated, they showed a quite robust relation (Pearson = 0.89 and R2 = 0.8). The relation of the AHIL with the aortic diameter revealed an intercept with weaker coefficients (Pearson = 0.48 and R2 = 0.23) but the overall regression was highly statistically significant (*p* < 0.001).
3. The difference between AHI and AHIL for risk categorization was statistically significant also in the “Control group”, a selected high-risk cohort with severely enlarged – not dissected - ascending aneurysm, with a *p*-value < 0.002.

An adequate risk stratification would be crucial to prevent acute aortic events, due to the high mortality rate, even if it is timely addressed. Although in Western countries the in-hospital mortality rate after surgery has decreased over the last decade, the incidence rate has increased for both sexes during this period (1–4). According to a secondary data analysis of Swiss DRG statistics, the adjusted in-hospital mortality rate for women decreased from 27.6% in 2009 to 18.5% in 2018, while for men, it decreased from 19.0% to 12.3% during the same period (14). Despite these improvements, AAAD is a life-threatening condition that needs prompt recognition and cardiological prevention (8, 9). Our data, along with literature data, showed that over 70% of patients operated on for AAAD had an aortic diameter below the threshold for prophylactic surgery (55 mm), and they could have been diagnosed before the event. This finding confirms the difficulty of diagnosing aneurysms of the ascending aorta, which are notoriously asymptomatic, and questions at the same time the adequacy of the current cut-off threshold for prophylactic surgery (11–14). The high proportion of dissected aortas below the threshold value indicates that a purely diameter-related approach often takes effect too late**.**

In addition to the single measurement of the aortic diameter for an indication of surgery, there are other assessment methods, such as the ASI and the AHI (Table 1). The ASI is strongly dependent on the body surface area (BSA) and thus body weight, and with extreme body constitution, the actual risk can either be overestimated (e.g., underweight) or underestimated (e.g., overweight or excessive muscle mass). As weight is included in the denominator, weight gain incorrectly reduces the calculated risk. ASI is a useful additional index but should not be used alone and must always be considered in conjunction with other clinical and morphological factors. The use of the AHI avoids this problem by eliminating the BSA from the calculation.

Recent evidence increasingly favours the inclusion of AAL as an independent risk factor for AAEs. Several studies have shown that aortic length, especially when indexed to height through the AHI, has a significant prognostic value beyond diameter alone (10–12, 15, 16). Wu et al. determined a critical threshold major or equal to 13 cm for AAL, which is strongly associated with an enhanced risk of AAEs. They showed that the combination of length and diameter in the AHI increases the predictive reliability (12). Thus, a prophylactic surgery should be considered at 11 cm. In a similar way, Heuts et al. confirmed that both aortic diameter and length independently predict the risk of AAAD (10, 16, 17). In addition, along with our published original data, Dagher et al. underlined the clinical importance of AHI in identifying candidates for prophylactic surgery, especially women who had impaired outcomes and survival, who might otherwise be overlooked using conventional diameter thresholds (7, 18, 19). The introduction of the AHIL represented an improvement, demonstrating that including aortic length in the AHI was a step towards a more accurate assessment of aortic risk (Figure 3). The combination of height, aortic diameter, and AAL can help to identify individuals at increased risk of AAEs. There were significantly less patients in the high- and severe-risk categories when the AAL was included. The different categorization between the AHI and the AHIL was highly significant in the control-group (*p* < 0.002). A different perspective on dissection is the distinction between potentially predictable and unpredictable AAAD. Preventable dissections typically occur in the presence of a dilated ascending aorta (AAo) and in such cases, tools like the AHI or AHIL could contribute to a better risk stratification and surgical planning. In contrast, non-preventable dissections may occur in patients without significant aortic dilation or with only a subtle enlargement that does not meet the established surgical thresholds and are therefore more difficult to anticipate. Atherosclerosis, like a penetrating aortic ulcer (PAU), undiagnosed genetic conditions (e.g., Marfan syndrome, Loeys-Dietz syndrome), or post-traumatic dissections are also considered unpredictable dissections as they may dissect in the absence of morphological warning signs (3). These situations highlight the limitations of size-based criteria alone and underline the need for more comprehensive risk models that integrate genetic, clinical, and morphological factors. In conclusion, a potential question for future research is a clinical validation whether the AHIL would be more accurate if the volume is taken into account in addition to the diameter and length of the ascending aorta. A volumetric index could enable more precise stratification by assessing localized bulges or asymmetric dilatations that linear measurements alone might miss.

### Limitations

As this is a retrospective observational study, it is limited by selection bias, incomplete or inaccurate data, and the inability to control for confounding factors. Also, the small number of patients in our control group represents a considerable limitation, as it reduces the statistical power and restricts the applicability of the results to the general population. The outcomes of this subgroup should be interpreted as exploratory. More, MRI measurements may show slight deviations compared to CT scans, as they are performed manually rather than automatically. The technique for calculating AHIL is highly sophisticated and is not available to every Institution. The AHIL threshold value for dissection could be slightly overestimated due to the dissective process itself and with the current data the manuscript provides a potentially useful exploratory morphologic index rather than a clinically validated risk score.

## Conclusion

The addition of ascending aortic length to the aortic height index provides a potentially useful exploratory morphologic index available to the cardiologic community, helping to discriminate individuals within the moderate risk for acute aortic events. Patients with an AHIL exceeding 9.7 cm/m should be referred to an Aortic Center for a proper multidisciplinary risk evaluation.

## Data Availability

The raw data supporting the conclusions of this article will be made available by the authors, without undue reservation.
